# Evaluating the Effectiveness of Mobile Health in Breast Cancer Care: A Systematic Review

**DOI:** 10.1093/oncolo/oyad217

**Published:** 2023-08-03

**Authors:** Madeleine Flaucher, Anastasiya Zakreuskaya, Michael Nissen, Alexander Mocker, Peter A Fasching, Matthias W Beckmann, Bjoern M Eskofier, Heike Leutheuser

**Affiliations:** Department Artificial Intelligence in Biomedical Engineering (AIBE), Machine Learning and Data Analytics Lab, Friedrich-Alexander-Universität Erlangen-Nürnberg, Erlangen, Germany; Department Artificial Intelligence in Biomedical Engineering (AIBE), Machine Learning and Data Analytics Lab, Friedrich-Alexander-Universität Erlangen-Nürnberg, Erlangen, Germany; Department Artificial Intelligence in Biomedical Engineering (AIBE), Machine Learning and Data Analytics Lab, Friedrich-Alexander-Universität Erlangen-Nürnberg, Erlangen, Germany; Department of Gynecology and Obstetrics, Erlangen University Hospital, Friedrich-Alexander-Universität Erlangen-Nürnberg, Erlangen, Germany; Department of Gynecology and Obstetrics, Erlangen University Hospital, Friedrich-Alexander-Universität Erlangen-Nürnberg, Erlangen, Germany; Department of Gynecology and Obstetrics, Erlangen University Hospital, Friedrich-Alexander-Universität Erlangen-Nürnberg, Erlangen, Germany; Department Artificial Intelligence in Biomedical Engineering (AIBE), Machine Learning and Data Analytics Lab, Friedrich-Alexander-Universität Erlangen-Nürnberg, Erlangen, Germany; Department Artificial Intelligence in Biomedical Engineering (AIBE), Machine Learning and Data Analytics Lab, Friedrich-Alexander-Universität Erlangen-Nürnberg, Erlangen, Germany

**Keywords:** digital health, mHealth, patient-reported outcome measures, wearable sensors, oncology

## Abstract

Breast cancer is affecting millions of people worldwide. If not appropriately handled, the side effects of different modalities of cancer treatment can negatively impact patients’ quality of life and cause treatment interruptions. In recent years, mobile health (mHealth) interventions have shown promising opportunities to support breast cancer care. Numerous studies implemented mobile health interventions aiming to support patients with breast cancer, for example, through physical activity promotion or educational content. Nonetheless, current literature reveals that real-world evidence for the actual benefits remains unclear. In this systematic review, we focus on analyzing the methodology used in recent studies to determine the effects of mHealth applications and wearable devices on the outcome of patients with breast cancer. We followed the PRISMA guideline for the selection, analysis, and reporting of relevant studies found in the databases of Medline, Scopus, Web of Science, and Cochrane Library. A total of 276 unique records were identified, and 20 studies met the inclusion criteria. Study quality was assessed with the Effective Public Health Practice Project (EPHPP) Quality Assessment Tool for Quantitative Studies. While many of the studies used standardized questionnaires as patient-reported outcome measures, there was minimal use of objective measurements, such as activity sensors. Adoption, drop-out rates, and usage behavior of users of the mobile health intervention were often not reported. Future work should clearly define the focus and desired outcome of mHealth interventions and select outcome measures accordingly. Greater transparency facilitates the interpretation of results and conclusions about the real-world evidence of mobile health in breast cancer care.

## Teaser

Mobile health (mHealth) interventions have shown promising opportunities to support breast cancer care. This review analyzes methodologies used in recent studies to determine the effects of mHealth applications and wearable devices on outcomes for patients with breast cancer.

**Implications for Practice**

Despite the growing use of mobile applications in breast cancer care, the effectiveness of such interventions remains inconclusive. With this systematic review, we aimed to analyze the current methodology used for the evaluation of mobile health interventions in breast cancer care. Our findings highlight the need for a standardized approach to assessing the impact of these interventions, enabling a clearer understanding of their effectiveness. By providing clear recommendations for future studies, we aim to facilitate informed decisions regarding the integration of mobile health interventions into breast cancer therapy.

## Introduction

Breast cancer is the most commonly diagnosed cancer worldwide.^1^ This corresponds to more than 2 million individuals receiving a breast cancer diagnosis annually. To date, early detection and therapy remain challenging, especially due to the differences in healthcare systems and socioeconomic resources.^2^ The variety of subgroups for breast cancer requires a wide range of therapeutic measures, including surgery, radiation, chemotherapy, endocrine therapy, and targeted therapies.^3,4^ These therapies can lead to a range of adverse effects that can impact the quality of life (QoL) of patients,^5,6^ such as nausea, fatigue, hair loss, and insomnia, among others.^6^ Furthermore, the diagnosis and treatment of cancer can be emotionally and mentally taxing. Many patients experience anxiety, depression, and other psychological difficulties.^7,8^

In the past years, there has been increasing interest in the use of mobile health (mHealth) applications,^9-12^ wearable devices,^13^ and telemedicine^14^ to support breast cancer management. Ample reviews aimed to summarize the evidence for the implementation of mHealth in breast cancer care. Suchodolska et al^15^ analyzed the effects of chemotherapy-related symptom reporting and management through mobile applications. While highlighting the feasibility and potential benefits, the authors acknowledge the limited significance of the results in the included studies. Within the systematic review of Jongerius et al,^10^ an overview of the measured impact of mHealth applications in empirical studies from 29 studies in breast cancer care was generated. The authors conclude that the evidence of mHealth interventions for patients with breast cancer is inconsistent and recommend further exploration, especially in the area of psychological interventions. This corresponds to the findings of Cruz et al,^16^ where the great potential is equally emphasized, but the actual benefit remains unclear.

All these systematic reviews have attempted to identify the current evidence regarding the use of mHealth interventions in breast cancer care. All of them come to a similar conclusion: a positive impact could exist, but the evidence remains limited in terms of its quality, consistency, and generalizability. Despite the large number of studies in the last years, it is unclear why evidence for the effectiveness of mHealth applications for breast cancer is still contradictory.

To address this knowledge gap, the objective of this systematic review is to analyze the methods used in recent studies for the evaluation of the effectiveness of mHealth interventions, such as smartphone applications and wearable sensors in breast cancer care. Through a detailed investigation of the study designs and methodologies, this review aims to identify barriers of current research to clear evidence. By acknowledging these limitations and identifying reasons that impede comparisons of different mHealth studies, we can work toward developing more standardized approaches for evaluating these interventions. This enables us to draw meaningful conclusions about the impact of mHealth in breast cancer care and facilitate the development of evidence-based interventions that can improve patient outcomes.

## Materials and Methods

### Search Strategy

This systematic review followed the PRISMA (Preferred Reporting Items for Systematic Reviews and Meta-analyzes) guidelines.^17^ A review protocol was drafted for internal use, and registration was not performed. The search was performed using the databases of Medline (Pubmed), Scopus, Web of Science, and Cochrane Library. To identify relevant records, the following search string was used; “(app OR application) AND (smartphone OR mobile OR wearable) AND (breast OR mammary) AND (cancer OR tumor).” In addition, results were filtered according to the language they were written in (only English) and the year (after January 1, 2018) they were published. All results were imported into a literature management tool to screen the records.

### Eligibility Criteria and Study Selection

The definition of the eligibility criteria was performed based on the PICO Statement.^18^ The following study characteristics were defined to be necessary for the inclusion of a study into this review: (1) the study population was composed of patients diagnosed with breast cancer; this includes patients during active treatment as well as during the follow up. (2) A mobile application or a wearable device was used by the patients themselves as an interventional support tool. (3) The outcome of interest should measure an impact on the patients with quantitative methods. (4) The article was published in English. (5) The article was published in a peer-reviewed journal after January 1, 2018. This time span was considered to represent the most recent and state-of-the-art research on mHealth interventions for breast cancer care to increase the relevance and applicability of the findings to current clinical practice.

All publications falling under one of the following aspects were excluded: (1) studies using an application only as a platform to provide questionnaires or similar, and (2) publications such as reviews, commentaries, conference abstracts, pilot studies, conference abstracts, and qualitative studies. The search process of this systematic review was concluded in January 2022. Two reviewers independently screened all found records. First, based on the title and abstract, afterward, a full text assessment was performed for the remaining publications. Disagreements about inclusion or exclusion were discussed among the reviewers after the screening.

### Data Extraction and Synthesis

The study characteristics were extracted by 2 reviewers independently into a custom structured excel sheet. The following items were collected: authors, publication year, journal, country, study design, sample size, mobile app or wearable, duration, outcome measures, and main results. Disagreements about the extracted data were discussed among the reviewers. Of every record, the author, year, journal, study design, population, sample size, country of conduction, outcome measures, mobile app or wearable features, main findings, and limitations were summarized. Based on the complexity of interventions and tools used for the outcome measures, every data collection method used in the study was categorized into one of the following topics: (1) quality of life; (2) symptom burden; (3) psychological side effects and symptoms; (4) physical activity and healthy lifestyle; (5) self-efficacy, self-perception, and social support; and (6) patient-relevant structural and procedural improvement. This topic includes positive care effects that can be indirectly related to the patient’s health, eg, improvement of health literacy, patient empowerment, reduction of therapy-related effort, and burden on patients and their families.

Furthermore, the main features of mobile apps and wearable devices were investigated in greater detail and categorized according to Mendiola et al^19^ into the following categories: (1) export of data, (2) gamification, (3) general education, (4) tailored education, (5) plan or orders, (6) reminder, (7) community forum, (8) addresses symptoms, (9) tracker, (10) social media, (11) usability, and (12) cost.

### Quality Assessment

To assess the methodological quality of the studies included in this review, the Effective Public Health Practice Project (EPHPP) Quality Assessment Tool for Quantitative Studies were used, as it enables a comparison of different study designs.^20,21^ Based on selection bias, study design, confounders, blinding, data collection methods, withdrawals, and dropouts, the quality is categorized as low, moderate, or strong. This classification was performed by 2 reviewers independently based on the supporting material of the tool for each publication. Disagreements were resolved with a third reviewer and consensus-based discussion.

## Results

### Study Selection

**Figure 1.**
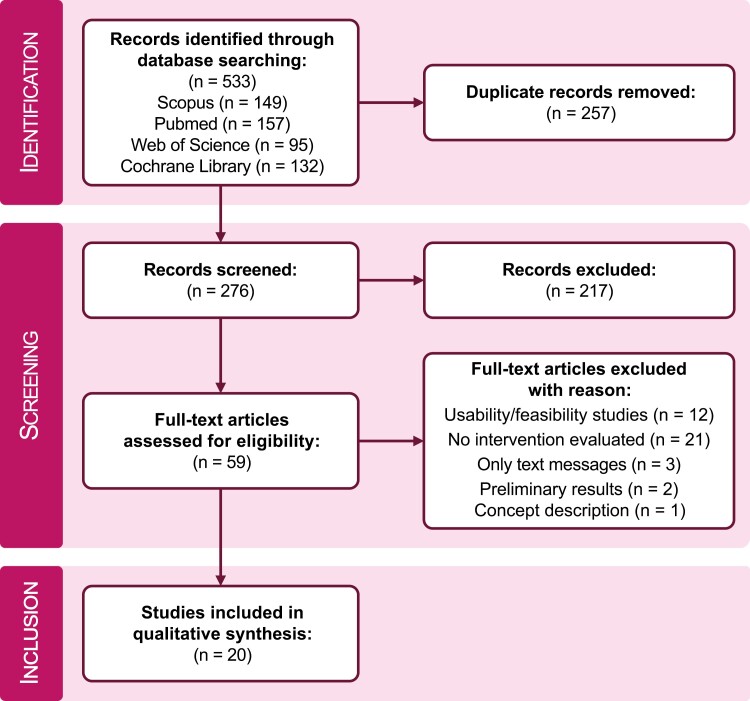
PRISMA flowchart representing the identification, screening, and inclusion phase of this systematic review.

In total, 533 records were identified in the extended bibliographic search. After removing the duplicates, the title and abstract of 276 records were screened. Sixty records met the inclusion criteria after screening the title and abstract and were eligible for the full-text assessment. Within this step, 12 records were excluded, as they were only usability or feasibility studies, 21 did not include the evaluation of an intervention, 3 only used text messages and no application, 2 described preliminary results, and 1 publication contained a concept description solely. Finally, 20 records met all inclusion criteria and were included in the synthesis of the results. The PRISMA flow diagram of the search and screening process is shown in Fig. 1. An overview of the eligible studies and their main characteristics is presented in Table 1.

**Table 1.** Overview of the studies included in this systematic review and their main characteristics.

Publication	App/wearable	Study design	Study objectives	Outcome measures	Quality rating	Country
Cairo et al^22^	Vida (App)	Non-randomized controlled trial	Evaluation of effects on diet adherence, physical activity, depression, fatigue, weight	GSLTPAQ, VAS-F, PHQ-2, BMI, weight, RYP	Weak	US
Chung et al^23^	WalkOn (App)	Non-randomized prospective clinical trial	Evaluation of effects on physical activity, distress	Daily steps, NCCN-DT	Weak	South Korea
Çinar **et al**^24^	Information Guide for BC Patients (App)	Randomized controlled trial	Evaluation of effects on QoL	FACT-ES, NCCN-DT	Weak	Turkey
Fjell **et al**^25^	Interaktor (App)	Randomized controlled trial	Evaluation of effects on symptom burden and QoL	MSAS, EORTC-QLQ-C30, number of reported symptoms	Moderate	Sweden
Ghanbari **et al**^26^	BCSzone (App)	Randomized controlled trial	Evaluation of effects on anxiety, self-esteem	STAI, RSES	Moderate	Iran
Grǎsǐc kuhar **et al**^27^	mPRO Mamma	Non-randomized controlled trial	Evaluation of effects on QoL, use of health resources	EORTC-QLQ-C30, EORTC-QLQ-BR23, number of doctor visits, hospitalizations	Moderate	Slovenia
Handa **et al**^28^	BPSS (App)	Randomized controlled trial	Evaluation of effectiveness as a tool to support patients during chemotherapy	HLS-14, HADS, number of reported side effects	Strong	Japan
Hou **et al**^29^	BCSMS (App)	Randomized controlled trial	Evaluation of effects on QoL	EORTC-QLQ-C30, EORTC-QLQ-BR23	Strong	Taiwan
Kim **et al**^30^	ILOVEBREAST (App)	Randomized controlled trial	Evaluation of effects on drug compliance, physical side effects, psychological conditions	K-MARS, BDI, STAI, WHOQOL-BREF, number of adverse events	Strong	South Korea
Kong **et al**^31^	Fitbit (wearable activity tracker)	Randomized controlled trial	Effectiveness to reinforce leisure-time physical activity	GPAQ, daily steps	Moderate	South Korea
Lozano-Lozano **et al**^32^	BENECA (App)	Pre-post study	Evaluation of effects on lifestyle, QoL, physical activity	EORTC-QLQ-C30, EAF (spanish), weight, body composition, accelerometer	Weak	Spain
Lozano-Lozano **et al**^33^	BENECA (App)	Randomized controlled trial	Evaluation of effects on QoL, functional outcomes in combination with a supervised rehabilitation program	EORTC-QLQ-C30, EORTC-QLQ-BR23, DASH, AROM, body composition	Strong	Spain
Öztürk and Kutlutürkan^34^	Msemptom (App)	Randomized controlled trial	Evaluation of effects on symptom control and QoL	MSAS; EORTC-QLQ-C30, EORTC-QLQ-BR23	Strong	Turkey
Park **et al**^35^	Pillsy (App) with smart pill bottle	Randomized controlled trial	Evaluation of effects on medication adherence with a smart pill bottle	CES-D, medication adherence, medication self-efficacy (custom)	Moderate	South Korea
Park **et al**^36^	Mobile App	Pre-post study	Evaluation of effects on menopausal symptoms, self-efficacy, and QoL	FACT-ES (menopausal symptoms), FACT-G, SESSM-B	Strong	South Korea
Rosen **et al**^37^	Headspace (App)	Randomized controlled trial	Evaluation of effects on QoL	FACT-B, MAAS	Moderate	US
Visser **et al**^38^	my-GMC (App)	Randomized controlled trial	Evaluation of effects on distress, empowerment	SCL-90, CWS, EORTC-QLQ-C30, EORTC-QLQ-BR23, MARS-5, CEQ (dutch)	Weak	Netherlands
Wyatt **et al**^39^	Web-application	Pre-post study	Evaluation of effects on decision-making confidence	Patient confidence in decision-making (custom)	Weak	US
Yu **et al**^40^	Management App	Retrospective observational study	Evaluation of effects on therapy adherence	Therapy adherence (custom)	Strong	China
Zhu **et al**^41^	BCS Care Breast (App)	Randomized controlled trial	Evaluation of effects on self-efficacy, social support, symptom distress, QoL, anxiety, depression	SICPA, MSPSS, MDASI, FACT-B, HADS	Moderate	China

Abbreviations: WHOQOL-BREF: World Health Organization Quality of Life; AROM: active range of motion; BDI: Beck Depression Inventory; BMI: body mass index; CEQ: Cancer Empowerment Questionnaire; CWS: Cancer Worry Scale; CES-D: Center for Epidemiologic Studies Depression Scale; DASH: disabilities of the arm, shoulder, and hand; EAF: Escala sobre actividad física; EORTC-QLQ: European Organization for Research and Treatment of Cancer-Quality of Life Questionnaire; FACT-B: Functional Assessment of Cancer Therapy-Breast; FACT-ES: Functional Assessment of Cancer Therapy-Endocrine Symptoms; FACT-G: Functional Assessment of Cancer Therapy-General; GAPQ: Global Physical Activity Questionnaire; GSLTPAQ: Godin-Shephard Leisure-Time Physical Activity Questionnaire; HADS: Hospital Anxiety and Depression Scale; K-MARS: Korean Version of the Medication Adherence Rating Scale; MDASI: MD Anderson Symptom Inventory; MARS-5: Medication Adherence Report Scale; MAAS: Mindful Attention Awareness Scale; MSAS: Memorial Symptom Assessment Scale; MSPSS: Multidimensional Scale of Perceived Social Support; NCCN-DT: NCCN distress thermometer; PHQ-2: Patient Health Questionnaire-2; QoL: Quality of life; RYP: rate your plate; RSES: Rosenberg Self-Esteem Scale; SESSM-B: Self-Efficacy Scale for Self-Management of Breast Cancer (SESSM-B); SICPA: Stanford Inventory of Cancer Patient Adjustment; STAI: State-Trait Anxiety Inventory; SCL-90: Symptom-Checklist-90; VAS-F: Visual Analogue Scale-Fatigue.

### Characteristics of Included Studies and Participants

The studies included in this systematic review were widely spread over the world. As shown in Table 1, most studies (*n* = 12, 60%) were conducted in Asia. Twenty-five percent were conducted in Europe and 15% in North America. Within the identified studies, 18 mobile applications and 1 wearable activity tracker (WAT) were used.

**Figure 2.**
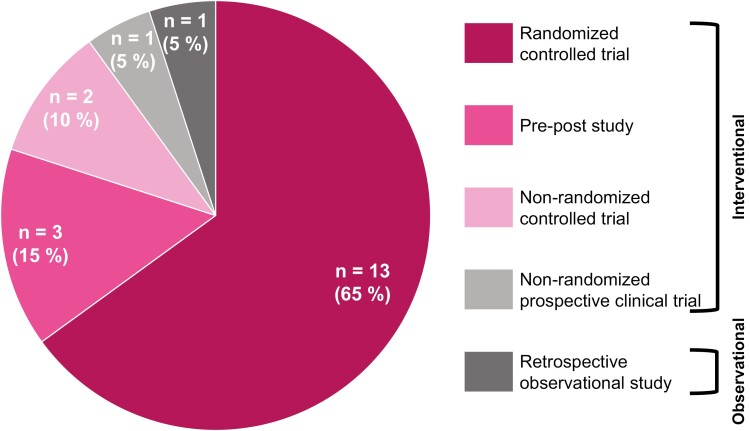
Overview of the different study designs and their interventional or observational character.

As shown in Fig. 2, all but one were interventional studies. One of the records used an observational design.^40^ An overview of the intervention period, sample size, and population of each study is shown in Table 2. The number of participants recruited for the studies varied in a range from 37^23^ to 4475^40^ participants. The intervention period ranged from 3 weeks^30^ to 6 months.^22,31^ In three publications,^27,39,40^ the duration of the intervention was only reported within a certain time span without further explanations about the actual duration of the intervention.

**Table 2.** Summary of the sample sizes, intervention period, and populations included into the studies analyzed in this review in alphabetical order.

Author	Sample size	Intervention period	Population
Cairo et al^22^	127	6 months	Patients with curative-intent (0-III) breast cancer
Chung et al^23^	37	12 weeks	Patients after surgery, without adjuvant chemotherapy
Çinar et al^24^	64	18 weeks	Patients with primary breast cancer, nonmetastatic, hormone receptor-positive, planned endocrine hormone therapy minimum 3 months
Fjell et al^25^	149	18 weeks	Recently diagnosed with planned neoadjuvant chemotherapy
Ghanbari et al^26^	77	4 weeks	Patients with nonmetastatic breast cancer
Grǎsǐc Kuhar et al^27^	91	Duration of treatment	Outpatients with early-stage breast cancer during systemic treatment
Handa et al^28^	95	12 weeks	Patients during anthracycline- or taxane-based chemotherapy
Hou et al^29^	100	12 weeks	Patients within 2 years after diagnosis (stages 0-III)
Kim et al^30^	72	3 weeks	Patients with metastatic breast cancer, cytotoxic chemotherapy planned
Kong et al^31^	118	6 months	Breast cancer patients with planned radiation therapy after surgery
Lozano-Lozano et al^32^	80	8 weeks	Patients with breast cancer stages I-IIIa
Lozano-Lozano et al^33^	78	8 weeks	Patients with breast cancer stages I-IIIa
Öztürk and Kutlutürkan^34^	57	6 weeks	Patients with breast cancer during chemotherapy
Park et al^35^	51	12 weeks	Patients with breast cancer stages I-III, after primary treatment with amenorrhea
Park et al^36^	57	4 weeks	Breast cancer patients during oral antiestrogen therapy
Rosen et al^37^	112	12 weeks	Within 5 years after diagnosis
Visser et al^38^	87	12 weeks	Breast cancer patients within 5 years since primary treatment
Wyatt et al^39^	255	1-4 weeks	Breast cancer patients (not further specified)
Yu et al^40^	4475	Approximately 2 years	Patients with planned multidisciplinary treatment discussion for adjuvant treatment
Zhu et al^41^	114	6 months	Patients with breast cancer (all stages) diagnosed within 3 to 8 weeks before

### Main Features of the mHealth Applications

**Figure 3.**
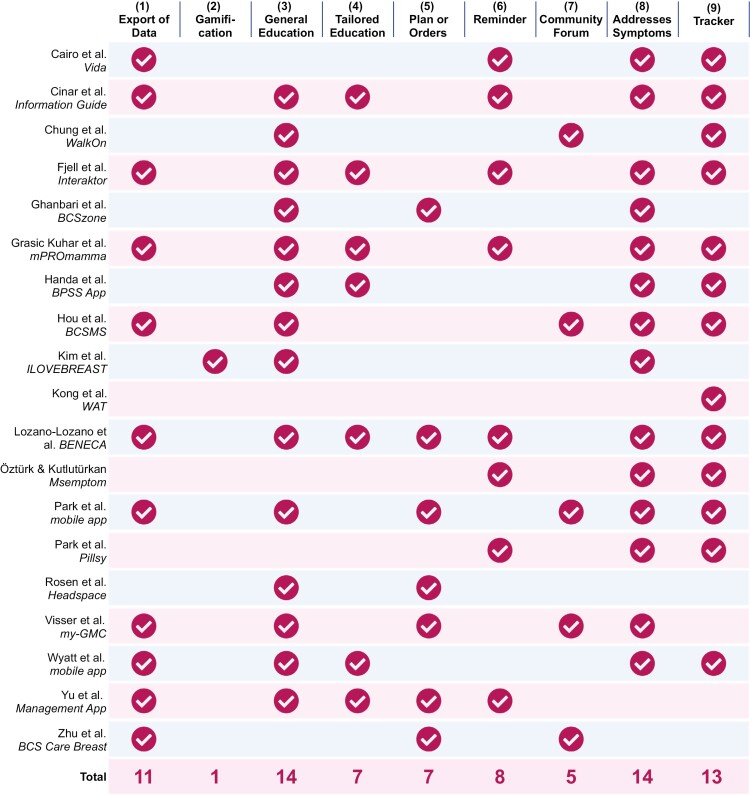
Overview of the respective features within the mobile applications and wearables, clustered according to Mendiola et al.^19^

Fig. 3 shows the distribution of the different features for all 18 applications and the wearable categorized according to Mendiola et al.^19^ The categories of Usability, Cost, and Social Media were omitted. The usability could not be evaluated due to missing access to most of the applications, and a usability analysis was not part of the studies. Because most apps were developed for research purposes and were, therefore, presumably made available to the participants free of charge, and cost was not analyzed. None of the analyzed concepts included a social media feature. Only 5 of the identified 19 mobile apps were available on the app stores of Google and Apple (Vida,^22^ WalkOn,^23^ Msemptom,^34^ Pillsy,^35^ Headspace^37^). None of them were specifically advertised for patients with breast cancer. While Pillsy and Msemptom are targeted at patients suffering from a disease or cancer in general, the other 3 apps are also designed for a broad audience to promote a healthy lifestyle. Within 3 studies,^36,39,40^ the application’s name was not mentioned, so it remains unclear whether these applications were published.

### Evaluation of User Behavior

Seven studies^23,24,26,27,29,33,34^ did not report whether any usage behavior was logged. Cairo et al^22^ only mentioned that in a follow-up consultation 6 months after the intervention, no participant used the app anymore. Visser et al,^38^ as well as Wyatt et al,^39^ analyzed self-reported usage of the application. The login frequency was reported in 3 of the studies.^28,37,41^ In the study of Rosen et al,^37^ the authors additionally had a look at the behavior within the app (performed lessons, date, and time of actions). Zhu et al^41^ also logged information about the duration of time spent in the app.

Park et al^36^ mentioned that login frequency and the duration of usage were tracked but did not report data about it. Two records^25,40^ evaluated whether the app was used. They reported that 23% of the participants never logged into the app. In the mobile game,^30^ the time the users spent playing was reported. Lozano-Lozano et al^32^ reported adoption, usage, and attrition rates in detail. This included the number of participants initially agreeing to use the application, actual active users, and dropouts over the whole study duration. In one study,^42^ usage frequency was indirectly measured through the medication tracked through the application. Finally, in the study incorporating a WAT,^31^ compliance was assessed by counting all days the WAT recorded more than zero steps.

### Quality Assessment

**Figure 4.**
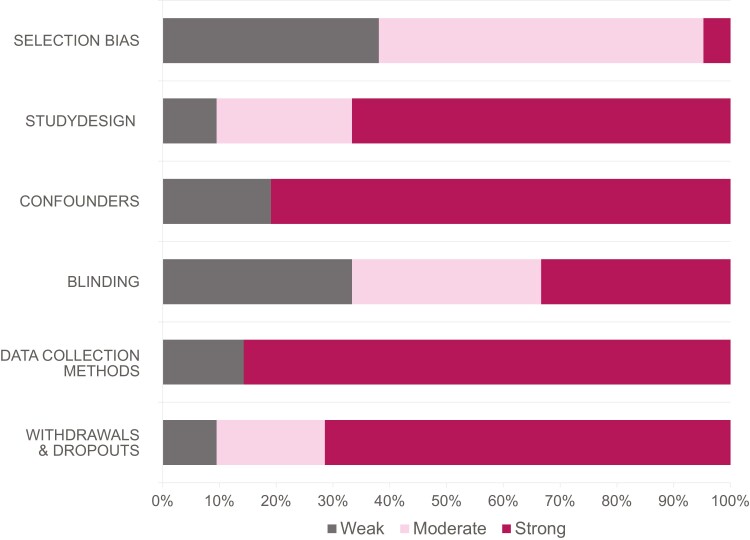
Overview of the quality ratings of the subcategories of the quality assessment with the (EPHPP) Quality Assessment Tool for Quantitative Studies.

The summarized results of the quality assessment of the studies can be found in Fig. 4. The result for the individual studies is listed in Table 1. Many studies were particularly weak in terms of the selection of study participants and the blinding. In contrast, most studies chose data collection methods that were shown to be both valid and reliable. Two of the studies^39,42^ used custom scales. Further details about the used outcome measures, their aims, and information about their validity and reliability will be analyzed in the following chapters.

### Outcome Measures

**Figure 5.**
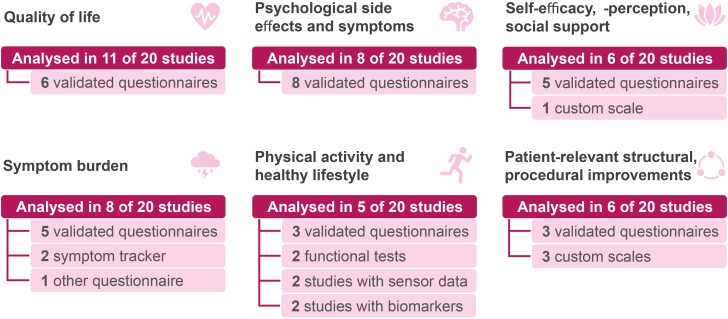
Overview of the categories of outcome measures used in the included studies and the type of measures applied.

The core aspect of the studies are the outcome measures used to show the impact of the mHealth application on patients with breast cancer. An overview of the findings is represented in Fig. 5.

#### Quality of Life

In 11 of the identified studies,^24,25,27,29,30,32-34,36,38,41^ one of the outcome measures were quality of life (QoL). In total, 6 different questionnaires were applied. Seven of the studies used the validated QoL of Cancer Patients questionnaires of the European Organization for Research and Treatment of Cancer (EORTC). Five of them^27,29,33,34,38^ used both the QLQ-C30, for patients with cancer in general,^43^ and the BR-23, specifically developed for patients with breast cancer.^44^ Two of the studies^25,32^ only used the QLQ-C30. While 5 of the studies^27,29,32,33,38^ reported the general QoL with these modular questionnaires, 2^25,34^ only reported specific subscales.

Three of the studies^24,36,41^ made use of validated questionnaires from the Facit Group.^45^ Zhu et al^41^ used the FACT-B,^46^ a questionnaire specifically designed for measuring the health-related QoL in patients with breast cancer. Park et al^36^ incorporated the FACT-G^47^ in their evaluation, a questionnaire for patients with cancer in general. The FACT-ES was applied in the study of Çinar et al,^24^ with its special focus on patients receiving endocrine treatments.^48^ To evaluate their mobile game, Kim et al^30^ used the WHOQOL-BREF^49^ of the World Health Organization.

### Symptom Burden

In 8^22,25,28,30,34,36,38,41^ of the studies, the effect of the intervention on symptom burden was analyzed. Five studies^22,25,34,36,41^ used validated questionnaires to measure symptom burden. Three studies^28,30,38^ did not use validated questionnaires, and 2 of them^28,30^ analyzed symptoms through patient-reported outcomes (PRO) in the application. In 2 of the studies,^25,34^ the Memorial Symptom Assessment Scale (MSAS)^50^ was used to assess comprehensive information on common cancer-related symptoms. Fjell et al^25^ additionally assessed the number of reported symptoms through their mobile app. To ­evaluate specific symptoms during endocrine therapy, Park et al^36^ used the subscale for menopausal symptoms of the FACT-ES.^48^

The MD Anderson Symptom Inventory (MDASI) was used in the study by Zhu et al^41^ to capture a variety of symptoms and their severity as experienced by the patients. Visser et al^38^ used the SCL-90, a Dutch questionnaire. Cairo et al^22^ solely assessed the subjective cancer-related fatigue and made use of the Visual Analog Scale-Fatigue (VAS-F).^51^

Two studies^28,30^ did not use questionnaires. Instead, the number of side effects that were reported through the mobile app was counted in one of the studies.^28^ Kim et al collected the number of adverse events according to the Common Terminology Criteria of Adverse Events (CTCAE).^30^

### Psychological Side Effects and Symptoms

Eight of the studies^22-24,26,28,30,38,41^ investigated the influence of their mHealth intervention on psychological side effects and symptoms. The most prominent evaluated aspects in this area were anxiety and depression,^26,28,30,41^ other aspects included distress,^23,24^ mindfulness,^37^ and fear of recurrence.^38^

Two of the studies^26,30^ used the Spielberger StateTrait Anxiety Inventory (STAI) to analyze the effects of their intervention on anxiety. Kim et al^30^ additionally applied the Beck Depression Inventory (BDI) to determine the severity of depressive symptoms. Park et al^35^ used the Center for Epidemiologic Studies Depression Scale (CES-D) for assessing depressive symptoms. To evaluate both anxiety and depression, the Hospital Anxiety and Depression Scale (HADS) was used in 2 of the publications.^28,41^

In 2 of the studies,^23,24^ distress among participants was measured using the Distress Thermometer of the National Comprehensive Cancer Network (NCCN-DT). Cairo et al^22^ applied the Patient Health Questionnaire-2 (PHQ-2)^52^ to capture the frequency of depressed mood and anhedonia of the study participants. One study^38^ assessed the fear of recurrence of patients using the Cancer Worry Scale (CWS).

Finally, Rosen et al^37^ used the Mindful Attention and Awareness Scale (MAAS) to capture the self-assessed dispositional mindfulness of participants in their study.

### Physical Activity and Healthy Lifestyle

In 5 of the studies,^22,23,31-33^ physical activity and a healthy lifestyle were investigated. Two of them^23,31^ made use of activity sensors built into the smartphone or wearable device as an indicator of physical activity. Two of the studies^32,33^ included functional tests and biomarkers. Three studies^22,32,33^ analyzed effects on the weight or body composition of participants. One study investigated the nutrition of participants.^22^

Kong et al^31^ included a WAT to accompany the mobile application. To assess physical activity, the authors analyzed the steps recorded through this tracker and additionally used the Korean version of the Global Physical Activity Questionnaire (GPAQ) of the World Health Organization.^53,54^ Chung et al^23^ included a step counter into their application. As an outcome measure for physical activity, the authors compared the total weekly steps of patients.

In the study of Cairo et al,^22^ the Godin-Shephard-Leisure-Time-Physical-Activity Questionnaire was used. Furthermore, body mass index (BMI) and weight were measured at baseline and postintervention as an indicator of lifestyle behavior. A nutrition assessment using the Rate Your Plate questionnaire, which assesses eating patterns, was also conducted.

Lozano-Lozano et al used a diverse set of outcome measures in their 2 studies.^32,33^ In their earlier study,^32^ accelerometer data of the smartphone, the weight, and body composition were analyzed. Complementary patients’ motivation for physical activity was investigated using the validated Spanish self-efficacy scale for physical activity (EAF).^55^ In the ­follow-up study, they focused on upper-limb functionality and body composition.^33^ The assessment of existing disabilities was performed with a Spanish version of the Disabilities of the Arm, Shoulder, and Hand (DASH) questionnaire. The active range of motion (AROM) of the shoulder was measured using a goniometer, and a digital handgrip was used to measure upper-body muscular strength.

### Self-Efficacy, Self-Perception, and Social Support

Self-efficacy was assessed in 2 different studies^36,41^ with 2 different questionnaires. One study^26^ investigated self-esteem, and 2 studies^38,41^ evaluated the social support of patients. One of the studies^39^ evaluated the confidence of patients in making decisions with a custom scale.

Park et al^36^ assessed the self-efficacy of their participants with the Self-Efficacy Scale for Self-Management for Breast Cancer (SESSM-B).^56^ The Stanford Inventory of Cancer Patient Adjustment (SICPA) was used in a Chinese version in one of the studies^41^ to investigate the self-efficacy of participants. Ghanbari et al^26^ made use of the Rosenberg Self-Esteem Scale (RSES) to investigate self-esteem or an individual’s overall sense of self-worth and personal value.^57^

Empowerment in patients with breast cancer was assessed with a Dutch version of the Cancer Empowerment Questionnaire (CEQ) in one of the studies.^38^ Zhu et al^41^ analyzed the social support of participants with the Chinese version of the Multidimensional Scale of Perceived Social Support (MSPSS). The confidence in making decisions was measured in one of the studies.^39^ Participants rated whether they prefer to make their own decisions or more doctor-guided decisions. In addition, participants ranked their confidence on a scale of 1 to 10.

### Patient-Relevant Structural and Procedural Improvement

As outcome measures for patient-relevant structural and procedural improvements, the studies investigated therapy and medication adherence,^30,35,38,40^ the number of visits to the doctor or hospital,^27^ health literacy,^28^ and medication self-efficacy.^35^

Three of the identified studies^30,35,38^ investigated the adherence to the medication, and one^40^ to the therapy in general. Medication adherence was measured using the Korean Version of the Medication Adherence Rating Scale (K-MARS)^58^ in the study of Kim et al^30^ Visser et al^38^ used the 5-item Medication Adherence Report Scale (MARS-5).^59^ The adherence to the therapy of the participants in the study of Yu et al^40^ was assessed through medical staff, usually the follow-up specialist or a nurse using predefined criteria. One of the studies^27^ evaluated the self-reported number of doctor visits and hospitalizations of patients during therapy.

One^28^ of the included studies investigated the health literacy of patients with the Japanese Health Literacy Scale (HLS-14).^60^ In the work of Park et al,^35^ medication self-efficacy was measured with a self-developed scale on which no details were provided.

## Discussion

Our findings show that current study designs are heterogeneous. This makes it difficult to derive direct comparisons and generate overarching evidence and conclusions. To support patients and healthcare providers in the adoption of mHealth applications, future developments should be guided through clear and consistent scientific guidelines or frameworks. A thorough understanding of the effectiveness of mHealth interventions is a prerequisite for the development in the field. At the same time, this will reduce unnecessary or even detrimental interventions.

### Targeted Outcomes of Specific Features Should Be Defined and Reported

The mHealth interventions analyzed in this review have a broad range of features (Fig. 3). This variety prohibits the assessment of how the effectiveness of these apps is related to their specific features. Some features may support positive outcomes, while others may not have an impact or may even cause negative outcomes.^61^ Our review reveals that descriptions of features, content, and interfaces were often limited. This hinders the understanding of how the different features contribute to the effectiveness of apps. It makes it challenging to compare the results across studies and, thereby, determine which features are most important for promoting positive outcomes and designing more effective interventions in the future. Open-source development of mHealth interventions may support the interpretation and reproducibility of results.

### Higher Transparency of Adoption, Adherence, and User Behavior Is Necessary

Thirty-five percent of the studies did not report any information about the user behavior. Without such information, the relation between the app and the effectiveness of the intervention cannot be adequately compared. This is especially important in light of short usage durations, the limited number of participants as well as, in some cases, wide inclusion criteria. This results in a study population with patients of many different stages and levels of severity and, thus needs, being included in a single study. Major issues with studies incorporating mHealth interventions are the high dropout rates and often limited adoption.^62-64^ Transparent reporting of acceptance, dropout, and engagement is essential to interpret the findings, as they may entail a bias in the final sample.

### Selection Biases Are Common

The quality assessment of the included studies revealed that, in general, the methodological quality was good. However, a common issue found among the studies was the presence of selection bias, mainly due to their single-center nature.

Furthermore, study participants were aware of the research questions in most of the studies. In contrast to these limitations in study quality, the data collection methods used were, in most cases, deemed to be valid and reliable. Finally, the reported information by studies was insufficient to determine the actual risk of bias.

### Heterogenous Outcome Measures Hinder Comparability

Our detailed analysis showed a large number of different outcome measures for single parameters. While the authors mostly chose validated measures, a lack of consistency in the measurement of outcomes makes it more difficult to draw meaningful conclusions about the effectiveness of mHealth applications. The development of a set of appropriate, standardized outcome measures that can be used consistently across studies reduces variability and improves comparability in the future.

### PRO Measures Should Be Complemented by Objective Measures

In all studies, questionnaires were the most commonly used method to assess PROs. In addition, researchers made use of reporting symptoms via their respective mobile apps. Previous studies have shown a significant improvement between such patient-reported symptoms and the overall survival rate of patients.^65,66^ As the ESMO Clinical Practice Guidelines highlight, using electronic systems to collect PRO measures can be beneficial for both patients and caregivers.^67^ mHealth interventions have a great potential to streamline the implementation of PROs in clinical practice by enabling remote data collection, longitudinal monitoring, and individual feedback systems. However, objective measurements such as physical activity tracking through smartphone sensors have been found to provide valuable insights that might not be captured through a questionnaire.^68^ Furthermore, expanding the scope of objective measurements to include physiological parameters such as heart rate or blood pressure can also lead to a deeper understanding of patient health and new opportunities in tailoring therapies. Future studies should explore and compare the effectiveness of different measurement methods through patient-centered strategies. This ensures the assessment of multidimensional parameters such as quality of life. In addition, the development of new objective measures needs to be considered to improve data quality and enhance the potential impact of mHealth interventions for breast cancer care.

### Limitations and Strengths

Some limitations apply to our work. Only a limited number of databases were searched and studies falling into the scope of this review might have been missed. Due to the strong heterogeneity of the included studies, a meta-analysis was not possible. Another limitation is the short time span the publications were chosen from. Due to large advances in the field of mHealth interventions in recent years, especially pushed forward through a global pandemic, we chose this time frame to be suitable.

The strength of this review is the systematic search process for eligible studies with strict criteria and the data extraction and analysis that was independently performed by 2 researchers. This limited the risk of errors during the synthesis process and quality assessment.

## Conclusion

The use of mHealth technologies in breast cancer care has gained increasing attention in recent years. Such technologies can improve patient outcomes, empower affected individuals, and finally reduce the burden of treatment on both patients and healthcare providers. However, the methodology and results of effectiveness evaluations of such interventions are still inconsistent. Consequently, a particular precaution is necessary due to the limited evidence. With this systematic review, we were able to pinpoint underlying factors that hinder the comparability of current mHealth applications applied in breast cancer care and provide guidelines to overcome these barriers in future work: Especially, the wide variety of outcome measures used to analyze single parameters (eg, QoL, symptom burden), the not-targeted app features and the lack of transparency in reporting applications and user behavior reveal the need for improvement to drive efficient and effective development. Therefore, for future studies, we recommend a clear focus on specific features and outcome parameters, the comprehensive provision of information on the functionality and content of mHealth applications, and the development of gold standards for the evaluation of effectiveness in the respective areas of mHealth applications in breast cancer care.

## Data Availability

No new data were generated or analyzed in support of this research.
